# Clinical impact of the Model for End Liver Disease (MELD) score on the presence of microvascular invasion and on the postoperative outcome in patients undergoing liver transplantation

**DOI:** 10.1590/0100-6991e-20212997

**Published:** 2021-12-10

**Authors:** BRAINNER CAMPOS BARBOSA, LEANDRO AUGUSTO RODRIGUES SANTOS, GUSTAVO HENRIQUE RASSI MAHAMED DAHER, DANIEL LAHAN MARTINS, SIMONE REGES PERALES, STEPHANIS KILARIS GALLANI, LARISSA BASTOS ELOY DA COSTA, EDUARDO ANDREAZZA DAL LAGO, IIKA DE FÁTIMA SANTANTA FERREIRA BOIN, NELSON MARCIO GOMES CASERTA, ELAINE CRISTINA DE ATAÍDE

**Affiliations:** 1 - PUC Goiás, Departamento de Medicina - Goiânia - GO - Brasil; 2 - Universidade Estadual De Campinas (UNICAMP), Hospital das Clínicas da Universidade Estadual de Campinas (HC UNICAMP) - Campinas - SP - Brasil

**Keywords:** Liver Neoplasms, Hepatic Cirrhosis, Liver transplant, Neoplasias Hepáticas, Cirrose Hepática, Transplante de Fígado

## Abstract

**Objective:**

to correlate clinical and epidemiological data with the pathological analysis of liver explants from patients undergoing liver transplantation for hetapocarcinoma in the UNICAMP HC and to verify whether the MELD and MELD-Na scores are reliable factors to predict a worse post-transplant prognosis.

**Methods:**

we studied liver transplants carried out between May 2010 and November 2017. After excluding 38 patients, we included 87, analyzing clinical and laboratory data for correlation with the outcome Microvascular Invasion (MVI). Subsequently, we computed the MELD and MELD-Na scores and performed a descriptive analysis of clinical and laboratory data and, finally, calculated ROC curves to assess the association between these laboratory parameters and mortality in these patients.

**Results:**

most patients were male (78.30%), with an average age of 58.53 years. Most liver diseases were caused by HCV (53.26%). We found no predictors for MVI among the laboratory parameters. The ROC curves for death identified the MELD score as the cutoff point with the highest combined sensitivity (90.91%) and specificity (37.50%), with a value of 10 points, whereas in the MELD-Na the cutoff point was 7 points, with a sensitivity of 90.91% and a specificity of 33.33%, both scores being significant.

**Conclusions:**

there were no reliable predictors of MVI between clinical, laboratory, and epidemiological variables. The MELD-Na score is more sensitive than the MELD one for predicting mortality in patients undergoing liver transplantation.

## RESUMO

**Objetivo:**

correlacionar dados clínicos e epidemiológicos com a análise patológica dos explantes hepáticos dos pacientes submetidos ao transplante hepático por hetapocarcinoma no HC da UNICAMP e verificar se os scores MELD e MELD-Na apresentam diferenças estatísticas para predizer pior prognóstico pós-transplante.

**Métodos:**

considerando os transplantes hepáticos ocorridos entre maio/2010 e novembro/2017, após excluir 38, 87 pacientes foram incluídos, analisando-se dados clínicos e laboratoriais desses pacientes para verificar se há independência entre esses e desfecho Invasão Microvascular (IMV). Posteriormente, realizou-se o cálculo do MELD e MELD-Na, a análise descritiva dos dados clínicos e laboratoriais e, por fim, curvas ROC para avaliar a associação entre esses parâmetros laboratoriais e o desfecho óbito nestes pacientes.

**Resultados:**

a maior parte dos pacientes foi do sexo masculino (78,30%), com idade, em média, de 58,53 anos. A maior parte das hepatopatias foi causada pelo VHC (53,26%). Não foi encontrado preditores para o desfecho IMV entre os parâmetros laboratoriais. As curvas ROC para o desfecho óbito identificaram o score MELD como ponto de corte de maior sensibilidade (90,91%) e especificidade (37,50%) simultâneas com o valor de 10 pontos, ao passo que no MELD-Na o ponto de corte foi 7 pontos, com sensibilidade de 90,91% e especificidade de 33,33%, sendo ambas as escalas significativas.

**Conclusões:**

não foram encontrados preditores para IMV entre critérios clínicos, laboratoriais e epidemiológicos. O score MELD-Na é mais sensível que o MELD para predizer mortalidade nos pacientes submetidos a transplante hepático.

## INTRODUCTION

The World Health Organization (WHO) estimates that in 2030, one million people will die from liver cancers worldwide^1^. Hepatocellular carcinoma (HCC), the most prevalent type of primary malignant liver tumors, is one of the biggest world health problems and has been increasing in incidence^2^ ^-^ ^4^. Hundreds of thousands of new diagnoses are made every year, and this neoplasm is responsible for 85% of cases in emerging countries, especially in those in which the hepatitis B virus (HBV) is endemic^1^ ^,^ ^5^ ^,^ ^6^. 

The development of HCC is related to risk factors that substantially increase the probability of occurrence of this cancer, such as hepatitis B virus (HBV), hepatitis C virus (HCV), alcoholic liver disease (ALD), non alcoholic fatty liver disease (NAFLD), and cirrhosis due to any etiology^5^ ^,^ ^7^. Generally, these diseases go through the stage of liver fibrosis before culminating in hepatocellular carcinoma, which does not exclude the possibility of direct evolution to HCC, though^8^. 

NAFLD has an estimated global prevalence of 25% and has become the most common cause of HCC, being among the second or third indication for liver transplantation^9^ ^,^ ^10^. Approximately one quarter of adults with NAFLD have non-alcoholic steatohepatitis (NASH), which usually leads to progressive liver fibrosis, cirrhosis, and eventually evolves to hepatocellular carcinoma^11^ ^-^ ^13^. 

Studies report a steady increase in the prevalence and incidence of NAFLD-related hepatocellular carcinoma. Soon there will likely be more cases of HCC related to NASH than to chronic hepatitis C. This will be possible because the treatment for the C virus is effective and can achieve viral eradication in almost all patients, while NASH is increasingly prevalent^14^ ^-^ ^16^. 

The treatment of choice for advanced liver failure is human liver transplantation, and this therapeutic modality is limited because the demand for this organ is greater than the supply. For this reason, there is a list of potential liver transplant recipients, the order being defined by clinical criteria that assess the risk of mortality in the transplant waiting list, such as the Model for End-Stage Liver Disease (MELD), adopted in Brazil in 2006^17^ ^-^ ^19^. 

Some histological features of HCC are directly related to the disease evolution, such as the occurrence of microvascular invasion (MVI). Several authors have shown that the presence of MVI was closely related with HCC recurrence, with worst prognosis and with short survival, thus limiting the therapeutic approach options (20). However, MVI is difficult to detect by imaging and laboratory methods, requiring histopathological evaluation of the liver explant, restricting the usefulness of this information in the preoperative period. Therefore, the attending physician may have limited information to consider available therapies^21^ ^-^ ^23^. 

Although the occurrence of MVI is still a poorly understood phenomenon, several studies have been carried out to understand the relationship with other clinical and epidemiological data^20^ ^,^ ^22^ ^,^ ^24^ ^,^ ^25^. Computed tomography (CT) analysis, for example, did not predict MVI and, to date, there are no other methods proven to be able to predict the occurrence of MVI in general^26^. Studies have shown that serum levels of some markers, such as alpha-fetoprotein (AFP) and Gamma GT, can predict the presence of MVI in patients with multiple liver nodules^22^. 

Therefore, predicting MVI or determining related clinical factors can help in the operative management and therapeutic planning, which can positively impact disease’s prognosis and patient survival. In this regard, the present study will be based on a retrospective analysis of patients undergoing liver transplantation, to determine whether the etiology of liver disease was able to predict the occurrence of microvascular invasion, in addition to discussing the clinical and epidemiological characteristics of all transplant recipients.

## GOALS

The aim of this study was to correlate clinical and epidemiological data with the pathological analysis of liver explants from all patients who underwent liver transplantation at the Hospital das Clínicas (HC) of the State University of Campinas (UNICAMP), in the period of May 2010 to November 2017, due to hepatocellular carcinoma caused by cirrhosis of any etiology. Then, we assessed the possible relationship between the cause of liver disease and other pre-clinical data with the outcomes microvascular invasion and death. In addition, we verified whether the MELD and MELD-Na scores presented differences in sensitivity/specificity to predict death in post-transplant patients..

## METHOD

This is a retrospective study that analyzed the medical records of all patients who underwent liver transplantation due to any disease in the period from May 2010 to November 2017, at the HC UNICAMP, in São Paulo - Brazil. We initially included 125 patients, excluding those whose records did not contain enough information for the present study. 

For the quantitative serum tests, we considered data with the closest date to the transplant, admitting a maximum of three months before the operation. We excluded 32 patients, whose records lacked information on the presence or absence of microvascular invasion, date of liver transplantation, date of death, history of smoking and alcoholism, history of diabetes mellitus and hypertension, weight, height, presence or absence of varicose esophageal veins, presence or absence of ascites, and dosage of the following biomarkers: aspartate aminotransferase (AST), alanine aminotransferase (ALT), alpha-fetoprotein, alkaline phosphatase, serum sodium, creatinine, gamma-glutamyltransferase (GGT), platelets, total bilirubin, serum albumin, and international normalized relationship (INR). 

With these data in hand, we calculated the MELD and MELD-Na scores for all patients. The calculation of MELD was performed using the formula: MELD = 10 * ((0.957 * ln [Creatinine]) + (0.378 * ln [Bilirubin]) + (1.12 * ln [INR])) + 6.43. The calculation of the MELD-Na used the formula: MELD-Na = MELD + 1.32 x (137 - Na) - [0033 x MELD* (137 - Na)], considering 125-137 mEq/L the correction value of the range for serum sodium, as determined by the United Network for Organ Sharing (UNOS) criteria. 

Subsequently, we performed a descriptive analysis of clinical and laboratory data, and we opted for measures of absolute and relative frequency for categorical variables. For continuous variables, we conducted a data probability distribution analysis; for those whose distribution was normal, we chose to use the mean and standard deviation as measures of central tendency and dispersion, respectively. For variables whose distribution was not normal, we preferred to summarize them by the median as a measure of central tendency and interquartile range as a measure of dispersion. To compare normally distributed continuous laboratory variables, we performed the Student’s t test, and the Kruskal-Wallis one for variables with non-normal distribution. 

We also conducted a correlation analysis to identify the independence between the available variables using the Spearman Correlation Test and included the independent variables in logistic regression models in the search for association, using the Odds Ratio for the outcomes “Microvascular invasion” and “Death” due to hepatocellular carcinoma. At first, we carried out a binary logistic regression analysis, and included associations that returned p-values <0.20 in the multivariate logistic regression model. In this, we applied progressive saturation by adding variables, observing the effects on the precision and on the adjustment of the other variables on the definitive analysis. 

Finally, using the Model End-stage Liver Disease (MELD) score and the variant that includes serum sodium values (MELD-Na), we constructed ROC curves by evaluating the association between the laboratory parameters of liver disease severity (used to calculate the score) and microvascular invasion/death in these patients. We thus aimed to establish the ideal cutoff point on the curve for the outcome as the one that shows the highest values of Sensitivity and Specificity simultaneously. 

We tabulated all data in Microsoft Excel© spreadsheets and performed the statistical analyzes using the Stata software version 16.0 (StataCorp. 2019. Stata Statistical Software: Release 16. College Station, TX: StataCorp LP), with a significance level of 5%. 

The study is in line with the required ethical precepts, having been previously submitted to the Ethics in Research Committee of UNICAMP, obtaining approval under opinion number 1,377,774.

## RESULTS

Table 1 shows the individual characteristics of the studied patients. Among the 93 patients, 72 (78.30%) were male, with an average age of 58.53 years (± 0.80 years) and 84 (91.3%) Caucasian. Forty-nine (53.26%) of the liver diseases were caused by the Hepatitis C virus, followed by alcoholic liver disease (17.39%). Even so, 65 patients (70.70%) had a history of alcohol intake, 49 (53.26%) were hypertensive, and the tumors were located mainly on the right side (80 - 66.67%) of the liver. Tumors had mean size of 3.05cm (± 1.98cm) and most were histological grade 2 (42 - 56.25%). Twenty-nine patients (31.52%) had microvascular invasion on histopathological analysis and 44 (47.83%) died, with a time to death of 27.5 days (± 92.5 days), and the main cause was sepsis, totaling 18 (40.91%) of the deaths (Table 2). 

**Table 1:** Epidemiological and clinical characteristics of patients undergoing hepatectomy for Hepatocellular Carcinoma.

Variable	n	%
Male sex	72	78.30
Age*	58.22 (36-81)	0.77
Weight (kg)	76.00	19.75
Height (cm)	169.00	11.75
BMI (kg/m^2^)	24.82	6.45
Race		
White	84	91.30
Brown	6	6.50
Black	2	2.20
Alcohol consumption	65	70.70
Diabetes Mellitus	28	30.4 3
Systemic Arterial Hypertension	49	53.26
Cause of Liver Disease		
Hepatitis C Virus	49	53.26
Hepatitis B Virus	6	6.52
Alcoholic Liver Disease	16	17.39
Cryptogenic cirrhosis	15	16.31
Mixed	6	6.52
Esophageal varices	55	59.80
Ascites	34	37.0
Multiple Nodes	36	39.10
Liver Location		
Posterior	3	2.50
Left	37	30.83
Right	80	66.67
Tumors’ histological grade (n=80)		
1	6	7.50
2	45	56.25
3	27	33.75
4	2	2.50
Nodule Size**	3.05	1.98
Microvascular Invasion	29	31.52
Time between Diagnosis and Treatment (days)**	68	113
Time to death (days)	27.50	192.50
n=42		
Survival**	6.00	4.75
n=44		
Death	44	47.83

*Mean and standard deviation **Median and Interquartile range

**Table 2:** Causes of death in patients undergoing hepatectomy for Hepatocellular Carcinoma.

Cause of death	n	%
Hepatocellular carcinoma	1	2.27
Hypovolemic shock	7	15.91
Unspecified shock	1	2.27
Shock secondary to liver failure	1	2.27
Primary graft dysfunction	4	9.09
Acute lung edema	1	2.27
Liver failure	1	2.27
Multiple organ failure	1	2.27
Acute liver failure	2	4.55
Respiratory failure	2	4.55
Head and neck neoplasm	1	2.27
Cardiorespiratory arrest	3	6.82
Sepsis	18	40.91
Post-reperfusion syndrome	1	2.27

Table 3 brings patients’ laboratory data, showing the mean albumin values below the lower reference limits in the group of patients without microvascular invasion (3.32 ± 0.94) and within the limits of reference in the group with MVI (3.66 ± 0.71). The median platelet count was also below the lower reference limits in both groups. Median alpha-fetoprotein, AST, and GGT values were above the upper limits of normality. The median MELD of patients was 12 (AI=7) in patients without MVI and 11 (5.5) in patients with MVI, with MELD-Na values being 11 (AI=11) and 8 (AI=6.5) for the same groups. There were no significant differences between groups for any of the evaluated parameters.

**Table 3:** Laboratory characteristics of patients undergoing histopathological analysis after hepatectomy for Hepatocellular Carcinoma.

Variable	Median (AI) without MVI	Median (AI) with MVI	p-value
Alpha-fetoprotein	11.05 (29.16)	11.3 (90.4)	0.994
Serum Albumin*	3.32 (0.94)	3.66 (0.71)	0.082
Serum bilirubin	1.68 (2.38)	0.96 (1.81)	0.078
Platelet count	65,000 (51,000)	109,000 (98,000)	0.078
Creatinine	0.86 (0.32)	0.85 (0.20)	0.798
ALT	65 (86)	55 (48.5)	0.248
AST	55 (89)	46 (48)	0.542
INR	1.4 (0.38)	1.18 (0.45)	0.090
GAMA-Gt	97 (126)	144 (224)	0.168
Serum sodium	140 (5)	139 (5)	0.478
Alkaline Phosphatase	109 (76)	125 (122)	0.085
MELD	12 (7)	11 (5.5)	0.159
MELD-Na	11 (11)	8 (6.5)	0.141

Table 4 shows the lack of difference between the causes of liver disease according to the outcome microvascular invasion, there being only an initial association with male sex and smoking, neither significant in the multivariate analysis. For the outcome death, in the bivariate analysis there was no association with any of the causes of liver disease. Male sex (p=0.012) and smoking (p=0.035) were identified as protective factors, and Systemic Arterial Hypertension, as a risk factor (p=0.037). However, only males remained in the multivariate analysis (OR=0.2189, 95% CI 0.0624 0.7683). 

**Table 4:** Preclinical factors associated with Microvascular Invasion and death in patients undergoing histopathological evaluation after hepatectomy for Hepatocellular Carcinoma.

	Bivariate logistic regression					Multivariate logistic regression			
	n (%)	Odds Ratio	p-value	95% Confidence Interval		Odds Ratio	p-value	95% Confidence Interval	
Outcome: Microvascular Invasion	29 (100.00)								
Non-viral liver diseasea	8 (27.59)	1							
Viral liver disease	19 (65.52)	1.5132	0.403	0.5738	3.9905				
Mixed liver disease	2 (06.90)	1.4375	0.779	0.1143	18.076				
Male sex	26 (89.66)	3.2029	0.083	0.8571	11.9685	2.6845	0.151	0.6986	10.3161
Smoking	23 (79.31)	2.5219	0.079	0.8999	7.0679	2.1545	0.154	0.7495	6.1936
Systemic Arterial Hypertension	14 (48.28)	0.7467	0.516	0.3092	1.8032				
Outcome: Death	44 (10 0.00)								
Non-viral liver diseasea	19 (43.18)	1							
Viral liver disease	22 (50.00)	0.4539	0.085	0.1846	1.1161	0.6129	0.331	0.2286	1.6436
Mixed liver disease	3 (06.82)	0.6316	0.608	0.1091	3.6561				
Male sex	29 (65.91)	0.2381	0.012	0.0781	0.7263	0.2189	0.018*	0.0624	0.7683
Smoking	24 (82.76)	0.3819	0.035	0.156	0.9351	0.5707	0.282	0.2053	1.5865
Systemic Arterial Hypertension	28 (96.55)	2.4469	0.037	1.0555	5.6723	2.2846	0.084	0.8957	5.8275

^a^Alcoholic and cryptogenic cause; *Collinearity statistics: tolerance >0.1 and VIF <10; Hosmer and Lemeshow Test: p=0.181; Nagelkerke’s R²=0.087.

Table 5 records the initial association between tumor size >5cm, total bilirubin >1.2mg/dL, platelet count < 00x109/L, Serum Albumin <3.5 or >5.5g/dL, GGT >130U/L, and the outcome microvascular invasion. However, no variable remained significant in the multivariate analysis. We identified no associations between the laboratory variables and death. 

**Table 5:** Clinical and laboratory factors associated with Microvascular Invasion and death in patients undergoing histopathological evaluation after hepatectomy for Hepatocellular Carcinoma.

	Bivariate logistic regression					Multivariate logistic regression*			
	n (%)	Odds Ratio	p-value	95% Confidence Interval		Odds Ratio	p-value	95% Confidence Interval	
Outcome: Microvascular Invasion	29 (100.00)								
Largest tumor >5cm	7 (24.14)	3.6909	0.040*	1.0594	12.8589	2.0562	0.316	0.5029	8.4078
Total Bilirubin >1.2mg/dL	14 (48.28)	0.4030	0.049*	0.1630	0.9968	0.6214	0.372	0.2184	1.76 80
Platelet count <100 x 109/L	14 (48.28)	0.3449	0.023*	0.1379	0.8625	0.5207	0.206	0.1895	1.4 3 07
Serum Albumin <3.5 or >5.5g/dL	10 (34.48)	0.4785	0.113*	0.1923	1.1904	0.7492	0.577	0.2718	2.0651
ALT >44U/L	16 (55.17)	0.9231	0.859	0.3807	2.2383				
GGT >130U/L	16 (55.17)	2.1405	0.095*	0.8758	5.2315	1.8856	0.187	0.7351	4.8365
Outcome: Death	44 (100.00)								
Largest tumor >5cm	8 (18.18)	2.4444	0.171*	0.6807	8.7786				
Total Bilirubin >1.2mg/dL	30 (68.18)	1.5306	0.329	0.6507	3.6005				
Platelet count <100 x109/L	31 (70.45)	1.5623	0.314	0.6556	3.7231				
Serum Albumin <3.5 or >5.5g/dL	23 (52.27)	1.5333	0.309	0.6725	3.4959				
GGT >130U/L	17 (38.64)	0.7441	0.486	0.3241	1.7082				
Microvascular Invasion	13 (29.55)	0.8387	0.696	0.3469	2,0280				

*Collinearity statistics: tolerance >0.1 and VIF <10; Test Hosmer and Lemeshow test: p=0.952; R² Nagelkerke =0.152.

Figure 2 depicts the curves constructed for the outcome death. For the MELD score the cutoff with greater sensitivity (90.91%) and specificity (37.50%) was 10 points, while for the MELD Na, the cutoff was 7 points, with a sensitivity of 90.91% and a specificity of 33.33%. Both scores were significant, with areas under the curves of 0.5727 for the MELD and 0.6032 for the MELD-Na. Deaths occurred on average after 27.5 days, ranging from one day to 53 months.

## DISCUSSION AND CONCLUSION

The development of theoretical-clinical models capable of predicting unfavorable outcomes helps in planning and managing the patient’s treatment. Although there are numerous methods able at predicting various illnesses, there is often a high level of difficulty in creating these tools. Great scientific production and distinct approaches to the same subject by different researchers are therefore needed. The present study aimed at correlating the clinical and laboratory information of patients undergoing liver transplantation due to HCC to verify whether there is a relationship between these data and MVI or death. Recently, other authors performed calculations using data obtained by multislice tomography and concluded that it was not possible to predict MVI, as Lahan et al.^26^, who concluded that quantitative tomographic analysis does not predict microvascular invasion. 

In this study, we observed that the epidemiological characteristics of patients agree with the national profile. In Brazil, about 70% of HCC cases are related to cirrhosis secondary to infection by viruses B or C^27^. In addition, there is a predominance of virus C over B nationally, with 54% and 16%, respectively^27^. In the present study, however, although most HCC cases were associated with virus C, the alcoholic cause is represented as the second most prevalent etiology, reaching 17%, while virus B was at 6% (Table 1). It is worth noting that the sample used in this study is smaller when compared to the study by Carrilho et al.^27^, which defined the Brazilian profile for HCC. This sample difference could justify the divergence between the second etiology of HCC being the alcoholic cause and not the B virus in our series. 

Currently, the lifestyle and eating habits of patients has drawn attention to metabolic changes. Soon the metabolic causes of cirrhosis may be more prevalent than the viral causes^28^. In addition, our study confirmed that alcoholism is also a matter of concern for hepatocellular carcinoma. 

 The result on the HCC etiologies was directly related to the lifestyle and/or comorbidities of the studied patients. After analyzing the medical records, we found that 70% of these patients reported chronic use of alcoholic beverages at some time prior to the transplant (alcoholic liver disease was the second most prevalent cause in this study). In addition, 30% had a diagnosis of diabetes mellitus and 53% were hypertensive, becoming apparent that most patients had some type of metabolic comorbidity, as recorded in Table 1. In addition, when compared to national data, patients in this study showed a higher percentage of co-infection by viruses B and C; while the country has a percentage of 2%, our patients displayed 6%. 

 The occurrence of liver disease due to an unknown (cryptogenic) cause, 16%, was also above the Brazilian prevalence, 3%. A possible reason could be NASH under-diagnosed cases. It is noteworthy that the study that described the Brazilian epidemiology dates to 2010^27^ . Therefore, we may be facing a changing trend in the Brazilian epidemiological profile, since the anti viral therapies are advancing, while patients’ lifestyle is not following suit^35^. 

Table 1 records the clinical characteristics of transplant recipients, 78% being men, with a mean age of 58 years (36-81). In addition, Table 4 shows that among all pre-clinical variables analyzed, male sex was a protective factor for the outcome death. Thus, although more men are affected by HCC (78%), a lower percentage died when compared with women. Females, however, were in a smaller number in our study, 21 (22%), which could justify this finding. On the other hand, the percentage of males (78%) was the same (78%) as in the study by Carrilho et al.^27^, who analyzed 1,363 patients with hepatocellular carcinoma in 2010. This information reveals that there was no trend towards a reduction in HCC in men, the rate remaining constant. Similarly, as seen in Table 4, we did not find any relationship between the analyzed parameters and the outcome microvascular invasion, not even male sex being significant. 

Table 2 lists the causes of death of all patients who died in the post-transplantation period. There were 14 different causes, the main one being sepsis, with approximately 41%, followed by hypovolemic shock, with approximately 16%. We believe these results were primarily due to liver transplant being a major operation and the use of immunosuppressives postoperatively. However, significant advances in surgical technique, in the immunosuppressive therapy, and anesthetic management have improved outcomes in the early and late postoperative period^29^. 

As for the quantitative serum tests, Zhao et al.^22^ concluded that the serum concentration of the biomarkers AFP >400mg/L, GGT >130U/L, tumor diameter >8cm, and tumors count >3 were predictors of MVI in patients diagnosed with Multinodular HCC. In our study, however, we were unable to reproduce these results, as we evaluated tumors larger than 5 centimeters, total bilirubin >1.2mg/dL, platelet count <100x109/L, serum albumin <3.5 or >5.5g/dL, ALT >4U/L or GGT >130U/L, as shown in Table 5. In summary, none of these variables had a significant relationship with microvascular invasion. We also carried out a generalized analysis of the sample, with the patients with one or multiple nodes in the same calculation, although 39% of these were multinodular, very similar to the proportion of 40% in the Yang study sample^30^. 

Importantly, other authors^20^ had already determined that microvascular invasion is a risk factor for severity and recurrence of HCC, although our study did not contemplate data on hepatocellular carcinoma recurrence. Moreover, when the tumor is close to five centimeters in diameter, the nodule usually begins to lose differentiation and may present microvascular invasion. Based on this, one could assume that the larger the nodule, the worse the patient’s prognosis, with a greater probability of recurrence^31^ ^,^ ^32^. However, we could not confirm this information, as, in addition to not finding a significant relationship between the presence of MVI and mortality, we were also unable to relate the nodule size to MVI, as shown in Table 5. This can be explained by the possible under-diagnosis of microvascular invasion that existed in the pathology evaluations, especially in older cohorts. 

**Figure 1:**
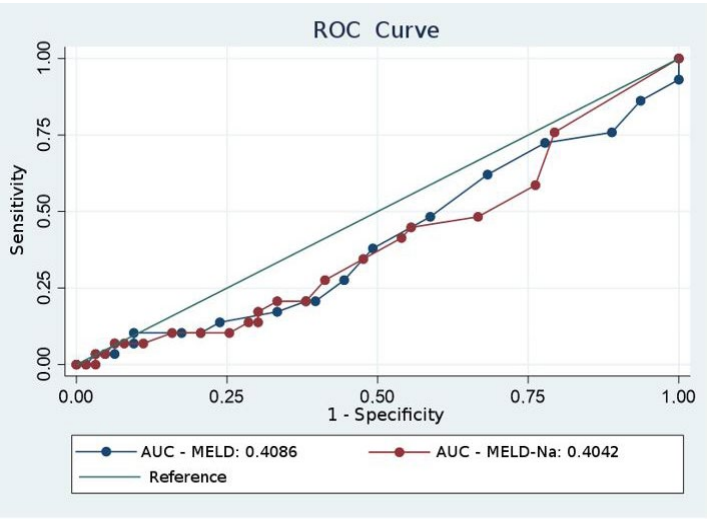
ROC curves and areas under the curve for MELD and MELD-Na score values versus microvascular invasion in patients with Hepatocellular Carcinoma. AUC: Area under the curve.

Initially, in Figure 1, the values of the areas under the ROC curves did not show any difference in sensitivity between the MELD and MELD-Na scores for microvascular invasion in patients with HCC. Thus, the curves for both scores were not significant - concluding that both scores could not be used as predictors of MVI. To date, we have not found any other study with a similar association. 

**Figure 2:**
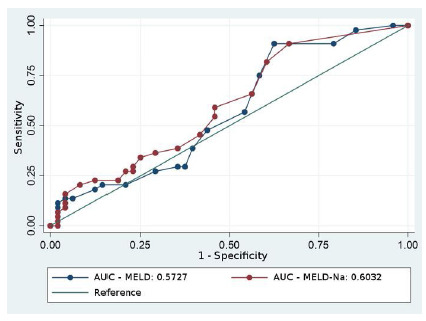
ROC curves and areas under the curve for MELD and MELD-Na score values versus death in patients with Hepatocellular Carcinoma. AUC: Area under the curve.

The ROC curve (Figure 2) showed a greater sensitivity of MELD-Na over MELD to determine death in patients with HCC, considering the same cutoff point for both. Other authors have shown that the MELD-Na score is more sensitive to determine severity and priority in the transplant queue^18^ ^,33^. More than that, we observed a close relationship between the MELD Na values and post-transplant death. From this analysis, we can infer that this score is also useful to assess postoperative prognosis. It is more sensitive than MELD alone, although both scores are statistically significant for this purpose under our analysis. Furthermore, the low specificity of this curve (37.50%) is explained by the large number of deaths resulting from non-hepatic causes, such as multiresistant germs and metabolic disorders. Our study is similar to that of Aranzana et al. ^19^ , who used the ROC curve for MELD values versus post-transplant survival in seven days, one month, three months, six months, one year, and two years, with 1,006 patients. The authors demonstrated that the score could serve as a significant predictor of post-transplant death. Similarly, Xun Luo et al.^34^ pointed out the functionality of the MELD-Na score in better predicting both mortality in the waiting list and survival. 

It is noteworthy that, despite having analyzed a single reference center, our study found data similar to the national profile and to other larger studies^27^ ^,^ ^30^. On the other hand, the retrospective analysis generated fragmentation and loss of information on body mass index (BMI) data, lifestyle habits, and brought uncertainties about the quantification and temporality of alcohol consumption. Thus, we identified the need for new methods and biomolecular parameters to analyze the tumor behavior and to define prognoses and strategies in the pre, intra, and postoperative period and also in the clinical follow-up.
